# Sir Henry Thompson

**Published:** 1904-05

**Authors:** 


Sir Henry Thompson, Bart., the distinguished English surgeon, died in London, April 18, 1904. He was born August 6, 1820, and was created baronet in 1899. He was the author of many scientific surgical papers, wrote two novels,—"Charley Kingston's

Aunt" and "All But,"—was a painter of no mean ability and. besides, was a student of astronomy.

